# Differences in clinicopathologic features and subtype distribution of invasive breast cancer between elderly and non-elderly women

**DOI:** 10.20407/fmj.2020-019

**Published:** 2020-10-10

**Authors:** Toshiaki Utsumi, Naomi Kobayashi, Kaori Ushimado, Makoto Kuroda

**Affiliations:** 1 Department of Breast Surgery, Fujita Health University, School of Medicine, Okazaki, Aichi, Japan; 2 Department of Breast Surgery, Nagoya Red Cross Hospital, Nagoya, Aichi, Japan; 3 Department of Diagnostic Pathology, Fujita Health University Okazaki Medical Center, Okazaki, Aichi, Japan

**Keywords:** Breast cancer, Elderly woman, Clinicopathologic characteristics, Subtype

## Abstract

**Objectives:**

This study aimed to investigate the clinicopathologic features and subtype distribution of invasive breast cancer in elderly women (≥70 years of age).

**Methods:**

This retrospective study of 1,130 women compared the clinicopathologic characteristics and subtype distribution of invasive breast cancer in elderly (≥70 years) versus non-elderly (<70 years) women. Tumors were classified into five distinct subtypes based on the immunohistochemistry status of estrogen receptor (ER), progesterone receptor (PR), Ki67, and human epidermal growth factor receptor 2 (HER2).

**Results:**

The two patient groups did not differ significantly regarding ER and HER2 status. Breast cancers in elderly women were more likely to have negative PR status (40.4% vs. 32.6%, *P*=0.033) and low Ki67 expression (62.0% vs. 54.4%, *P*=0.047) than those in non-elderly women. Elderly women were less likely to undergo axillary lymph node dissection and axillary surgery (*P*<0.001). Consequently, unknown node status was more common in elderly women than non-elderly women (11.1% vs. 1.4%, respectively, *P*<0.001), while node involvement was less common in elderly women than non-elderly women (26.9% vs. 37.7%, respectively, *P*<0.001). There was no significant difference in the distribution of subtypes between the two groups.

**Conclusions:**

Breast cancers in elderly women were less frequently node positive and more frequently PR negative and with low Ki67 expression than those in non-elderly women. Moreover, there was no difference in subtype distribution between the two age groups.

## Introduction

Breast cancer is the most common cause of cancer-related death in women in many countries.^1^ The incidence of breast cancer is lower in Japanese women than Western women,^2^ but it has been increasing in Japan,^3^ including in elderly women.^3^ Some studies have shown that breast cancer in elderly women is more indolent and less aggressive and proliferative than in breast cancer in non-elderly women,^4–6^ although one study presented conflicting data.^7^

Microarrays and related technologies have provided new genetic approaches for investigating the complex clinical issues related to breast cancer outcome.^8,9^ Studies using microarray analyses have shown that breast cancer is a heterogeneous disease with different subtypes that are characterized by distinct aberrations at the molecular level. According to gene expression studies, breast cancer can be classified into at least five distinct subtypes: luminal A, luminal B, human epidermal receptor type 2 (HER2) overexpressing, basal-like, and normal-like.^8–11^ Differences in gene expression patterns have been significantly correlated with differences in clinical outcomes.^9^

Studies have shown that protein expression can serve as a surrogate for genomic profiles when classifying breast cancer into subtypes with distinct biological characteristics and clinical outcomes.^12,13^ Classification of protein expression subtypes instead of molecular subtypes is now widely used in daily clinical practice because of the feasibility of protein expression assessment. A statement of the St. Gallen International Expert Consensus includes treatment algorithms based on the classification of breast cancer subtypes by immunohistochemistry findings for estrogen receptor (ER), progesterone receptor (PR), HER2, and Ki67 expression.^14,15^ Although breast cancer is a heterogeneous assembly of diseases, it can be clinically divided by hormone receptor, HER2, and Ki67 expression to guide therapeutic interventions. ER and HER2 are well-established therapeutic targets. Endocrine therapy is a standard of care for patients with ER-positive disease.^10,11^ Anti-HER2 therapy combined with chemotherapy is now widely accepted as a standard of care for patients with HER2-positive tumors more than 1 cm in size.^10,16^

Breast cancer subtypes have been well investigated in younger women,^17–19^ but only one such study has focused on subtypes in elderly women.^20^ In this study, we examined the clinicopathologic characteristics and subtype distribution of invasive breast cancer in elderly versus non-elderly women in a single institution.

## Methods

### Subjects

Between 2003 and 2014, a total of 1,704 patients with breast cancer were treated at Fujita Health University Hospital. Patients with stage IV, occult, noninvasive, or bilateral disease were excluded from this study. Male patients with breast cancer and patients lost to follow-up immediately after surgery were also excluded. A total of 1,130 women with invasive breast cancer were finally enrolled and were divided into two groups: elderly, defined as patients aged ≥70 years, and non-elderly, defined as patients aged <70 years. Histologic grade was assessed according to the Bloom and Richardson classification system.^21^ We investigated the relationship between clinicopathological factors (stage, T stage, pathological node status, histological grade, ER status, PR status, HER2 status, subtype distribution, types of operation, chemotherapy, endocrine therapy, and anti-HER2 therapy) and the two age groups. We also investigated distant disease-free survival (DDFS) and overall survival (OS) in the two age groups. This retrospective study was approved by the Ethics Committee of Fujita Health University (No. HM16-138).

### Immunohistochemistry

Immunohistochemical methods were described previously.^22–24^ Immunohistochemical staining for ER and PR was carried out using the SP1 and 1E2 staining systems (Ventana Medical, Tucson, AZ, USA), respectively. Positive ER or PR status was defined as ≥1% nuclear staining. Immunohistochemical assays for HER2 were performed using the Pathway anti-HER2/neu test (Ventana Medical). Fluorescence in situ hybridization as performed using the PathVysion HER-2 DNA probe kit (Abbott France SAS, Rungis, France). An immunohistochemistry score of 3+ or fluorescence in situ hybridization amplification was defined as positive. Ki67 staining was performed using the monoclonal antibody MIB-1 (Dako, Glostrup, Denmark). The Ki67 labeling index was categorized as low (<20%) or high (≥20%). All markers were assessed with blinding to the clinical data.

### Breast cancer subtype classification

Tumors were classified into five distinct subtypes based on the status of ER, PR, Ki67, and HER2 immunohistochemistry results: luminal A (ER+ and/or PR+, HER2–, and low Ki67), luminal B (HER2–) (ER+ and/or PR+, HER2–, and high Ki67), luminal B (HER2+) subtype (ER+ and/or PR+ and HER2+), HER2 overexpressing (ER–, PR–, and HER2+), and triple negative (ER–, PR–, and HER2–).

### DDFS and OS by age group

The events considered in our study of DDFS were first distant recurrence and death from any cause. DDFS was calculated from the date of diagnosis to the date of distant metastasis or death. OS was calculated from the date of diagnosis to the date of death from any cause.^25^

### Statistical analysis

Statistical analysis was performed using SPSS 22.0 software (IBM Corp., Armonk, NY, USA). The chi-square test was performed for contingency table analysis. Survival curves were generated using the Kaplan–Meier method.^26^ Survival comparisons were made using the log-rank test.

## Results

### Pathologic tumor characteristics of study patients

Table 1 shows the clinical profiles of the 1,130 women included in this study. Of the 1,130 patients, 208 (18.4%) were elderly and 922 (81.6%) were non-elderly women. Data on pathologic node status were missing for 36 women, 23 of whom were elderly and 13 of whom were non-elderly; axillary surgery was performed in six of the 13 non-elderly women, while surgery was not performed in any of the 23 elderly women. Seven non-elderly women underwent neoadjuvant chemotherapy, and in six of these patients, no information was available regarding pathologic node status before neoadjuvant chemotherapy. The remaining patient had no pathologic node involvement after neoadjuvant chemotherapy and no evidence of negative lymph node status before neoadjuvant chemotherapy. In total, 13 non-elderly patients had unknown node status. Consequently, there was a significant difference between the two age groups in pathologic node status; a higher proportion of breast cancers had unknown node status in elderly women than in non-elderly women (unknown node status, 11.1% vs. 1.4%, respectively, *P*<0.001) and a lower proportion of breast cancers had node involvement in elderly women than in non-elderly women (node positive, 26.9% vs. 37.7%, respectively).

No data on histologic grade were available for 11 tumors in elderly patients and 22 tumors in non-elderly patients. There was no significant difference in histologic grades between the two age groups.

### Biological markers and immunohistochemical breast cancer subtypes

Table 2 shows the biological profiles and the distribution of breast cancer subtypes in the 1,130 patients. There were no significant differences in ER or HER2 status between the two age groups. However, breast cancers in elderly women were more likely to have negative PR status (40.4% vs. 32.6%, *P*=0.033) and low Ki67 expression (62.0% vs. 54.4%, *P*=0.047).

Of the 1,130 tumors, 48.4% were luminal A, 23.0% were luminal B (HER2–), 7.5% were luminal B (HER2+), 7.1% were HER2 overexpressing, and 14.0% were triple negative subtype. There was no significant difference in the distribution of subtypes between the two age groups.

### Patient treatments

We investigated the relationship between surgical treatment and age group. There were no significant differences between the two age groups in the proportion of patients treated with breast surgery. Axillary surgery and axillary lymph node dissection were both less common in elderly women than non-elderly women (*P*<0.001) (Table 3). We also investigated the relationship between medical treatment and age group. Chemotherapy was administered to 19.2% of elderly women and 52.4% of non-elderly women (*P*<0.001) (Table 3). Anti-HER2 therapy was administered to 6.7% of elderly women and 12.1% of non-elderly women (*P*=0.025). There were no significant differences in the rates of endocrine therapy between the two age groups.

### DDFS and OS by age group

**Figure 1:**
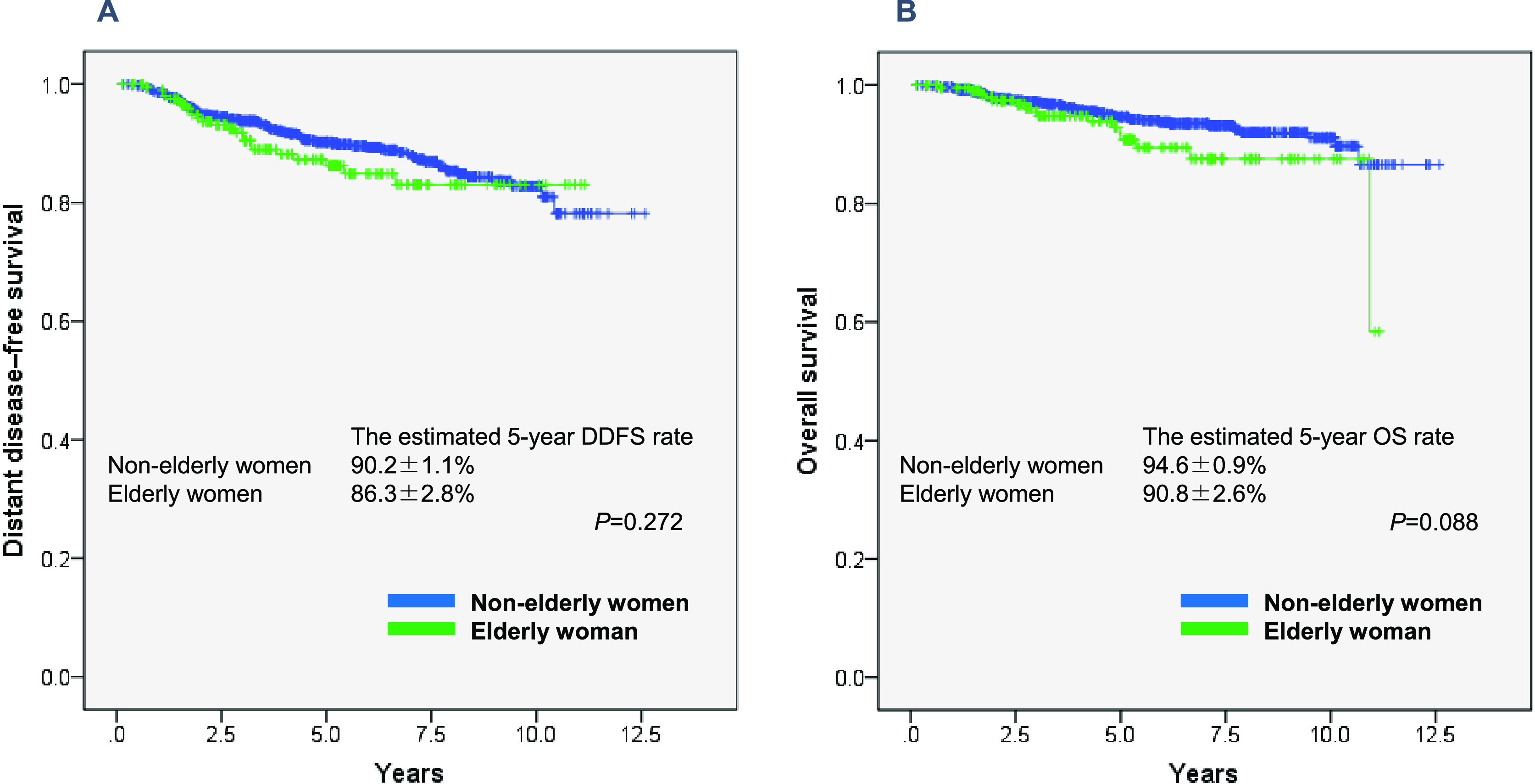
Distant disease–free survival and overall survival in 1,130 women with breast cancer (A) Distant disease–free survival and (B) overall survival by age group.

The overall median follow-up was 5.10 years [4.21 (range: 0.15–11.16) years for elderly patients and 5.23 (range: 0.15–12.59) years for non-elderly patients]. There was no significant difference in DDFS and OS between the two age groups (Figure 1). The estimated 5-year DDFS rate was 90.2±1.1% for breast cancer in non-elderly women and 86.3±2.8% in elderly women. The estimated 5-year OS rate was 94.6±0.9% in non-elderly women and 90.8±2.6% in elderly women.

## Discussion

There have been few guidelines for the management of elderly women with breast cancer. A main reason is the lack of strong evidence based on randomized controlled trials on the efficacy and safety of adjuvant therapy in this population. Therefore, oncologists must often make treatment decisions in the face of relative uncertainty. To better understand the characteristics of breast cancer in elderly women, we reviewed the clinicopathologic characteristics and subtype distribution of invasive breast cancer in elderly versus non-elderly patients in our institution.

The peak age at diagnosis for breast cancer is between 60 and 70 years old in Western countries, but between 40 and 50 years old in Asian countries.^27^ In studies of women with breast cancer, the definition of “elderly” varies; previous studies have used cutoff ages ranging from 67 to 80 years,^28–33^ while our study defined elderly as age ≥70 years. In Japan, 19.3% of women with breast cancer diagnosed between 2004 and 2009 were aged ≥70 years according to the Registration Committee of the Japan Breast Cancer Society.^34^ The proportion in our study was similar, at 18.4%.

Previous studies reported that breast cancer in elderly women is more indolent with less aggressive and proliferative characteristics than breast cancers in younger women.^4–6^ However, this issue remains controversial. In a study by Kim et al. in South Korea, breast cancer in elderly Korean women had more aggressive clinicopathological and biological characteristics than in Korean women of all ages or elderly women globally.^7^ We found that breast cancers in elderly women were less frequently node positive and more frequently PR negative and with low Ki67 expression than those in non-elderly women. Our data regarding Ki67 expression is consistent with the findings of Eppenberger-Castori et al.^5^ Some studies reported that tumors with higher expression of Ki67 demonstrated more lymph node involvement.^35,36^ These results suggest that the lower Ki67 expression in elderly women might result in a reduced rate of lymph node involvement compared with non-elderly women. Why breast cancer in elderly women was more likely to have low expression of Ki67, a proliferation marker, is unclear. This finding might be ascribed to differences in plasma estradiol levels between the two age groups. Estradiol has been shown to enhance ER-induced proliferation of MCF-7 breast cancer cells by stimulating expression of Ki67.^37^ As the rate of ER positivity was not different between the two groups in our study, and the non-elderly group includes premenopausal women whose plasma estradiol levels are higher than postmenopausal women, the elderly group could have low Ki67 expression. No previous studies have demonstrated that elderly women have a lower incidence of PR-negative breast cancer than younger women. This finding of the present study should be carefully interpreted due to the small sample size, and further confirmation is required in a larger series.

We found that there was no significant difference in the distribution of breast cancer subtypes in elderly versus non-elderly women. This contrasts with the results of Jenkins et al.,^17^ who performed an analysis using microarray datasets. This discrepancy might be caused by the use of different subtype definitions, sample sizes, or study populations.

Our results did not indicate any significant differences in DDFS or OS between the two age groups. Tumors in elderly women were less likely to involve the lymph nodes and more likely to have low Ki67 expression than those in non-elderly women, and thus the elderly patients had better prognostic factors. Chemotherapy was used less frequently in elderly women compared with non-elderly women. Prognostic prediction has historically been influenced by the anatomical extent of the tumor, as reflected by stage classification, but it has become clearer that tumor biology is more relevant to prognosis than tumor size.^38^ Breast cancer is now considered a heterogeneous condition comprising different subtypes with varying clinicopathologic features, outcomes, and responses to systemic therapy. The present study showed no significant difference in subtype distribution between elderly versus non-elderly women, which may be related to the similar outcomes between the two age groups. In our cohort, there was no influence of non-cancer-related death on OS in the elderly patients. The median follow-up was 1 year longer in the non-elderly patients than in the elderly patients. If the median follow-up had been the same, our results might be different.

A meta-analysis of the Early Breast Cancer Trialists’ Collaborative Group for the efficacy of chemotherapy did not show a benefit for chemotherapy in breast cancer patients older than 70 years of age.^39^ Age itself should not be an exclusion factor for a standard of care, but some elderly patients likely cannot tolerate standard therapies. Decisions about treatment in the elderly may be influenced by a number of factors including comorbidities, performance status, and other conditions that might cause the potential risks of treatment to outweigh the benefits. The precise assessment of the patient, taking into consideration their functional status, performance status, life expectancy, wishes, and the risks and benefit of each treatment, is considered an important issue in patient management and choosing the appropriate therapy for each patient.

Our study has several limitations. First, this was a retrospective, single-center study and therefore may have been prone to selection bias. Second, the number of elderly patients was small. Because relatively small studies might not provide definitive results, the results must be interpreted with caution. A larger observational series might yield additional data. Third, comorbidities should have been analyzed because these are more common in the elderly, but these data were not precisely recorded in all medical records. Despite these limitations, our study has several strengths. First, this study analyzed precise data regarding pathological factors and clinical outcomes in both age groups. Second, this study addressed the relationship between breast cancer subtypes and age, which is now widely thought to be an important issue in the field of breast cancer.

In conclusion, breast cancers in elderly women were less frequently node positive and more likely to be PR negative and to have low Ki67 expression than those in non-elderly women. Moreover, there were no differences in subtype distribution between the two age groups. Further studies with a larger number of patients are recommended to validate our findings.

## Figures and Tables

**Table1:** Tumor pathological characteristics

	Elderly patients	Non-elderly patients	*P* value
Number of patients	208	922	
T stage			
T1	94 (45.2%)	455 (49.3%)	
T2	95 (45.7%)	390 (42.3%)	
T3	4 (1.9%)	35 (3.8%)	
T4	15 (7.2%)	42 (4.6%)	0.161
Pathologic node status			
Negative	129 (62.0%)	561 (60.8%)	
Positive	56 (26.9%)	348 (37.7%)	
Unknown	23 (11.1%)	13 (1.4%)	<0.001
Stage			
I	92 (44.2%)	423 (45.9%)	
IIA	71 (34.1%)	306 (33.2%)	
IIB	25 (12.0%)	115 (12.5%)	
IIIA	5 (2.4%)	29 (3.1%)	
IIIB	14 (6.7%)	39 (4.2%)	
IIIC	1 (0.5%)	10 (1.1%)	0.641
Histologic grade			
1	56 (26.9%)	256 (27.8%)	
2	108 (51.9%)	492 (53.4%)	
3	33 (15.9%)	152 (16.5%)	
Unknown	11 (5.3%)	22 (2.4%)	0.169

**Table2:** Biological profiles and subtypes

	Elderly patients	Non-elderly patients	*P* value
ER			
Negative	45 (21.6%)	210 (22.8%)	
Positive	163 (78.4%)	712 (77.2%)	0.722
PR			
Negative	84 (40.4%)	301 (32.6%)	
Positive	124 (59.6%)	621 (67.4%)	0.033
HER2			
Negative	182 (87.5%)	783 (84.9%)	
Positive	26 (12.5%)	139 (15.1%)	0.342
Ki67			
Low (<20%)	129 (62.0%)	502 (54.4%)	
High (≥20%)	79 (38.0%)	420 (45.6%)	0.047
Subtype			
Luminal A	110 (52.9%)	437 (47.4%)	
Luminal B (HER2–)	42 (20.2%)	218 (23.6%)	
Luminal B (HER2+)	12 (5.8%)	73 (7.9%)	
HER2 overexpressing	14 (6.7%)	66 (7.2%)	
Triple negative	30 (14.4%)	128 (13.9%)	0.549

Abbreviations: ER, estrogen receptor; PR, progesterone receptor; HER2, human epidermal growth factor receptor 2

**Table3:** Patient treatments

	Elderly patients	Non-elderly patients	*P* value
Number of patients	208	922	
Breast surgery			
No breast surgery	0 (0%)	2 (0.2%)	
Breast-conserving surgery	111 (53.4%)	559 (60.6%)	
Mastectomy	97 (46.6%)	361 (39.2%)	0.116
Axillary surgery			
No axillary surgery	23 (11.1%)	6 (0.7%)	
ALND±SNB	63 (30.3%)	377 (40.9%)	
SNB	122 (58.7%)	539 (58.5%)	<0.001
Adjuvant and/or neoadjuvant chemotherapy			
Not given	168 (80.8%)	439 (47.6%)	
Given	40 (19.2%)	483 (52.4%)	<0.001
Adjuvant and/or neoadjuvant endocrine therapy			
Not given	42 (20.2%)	210 (22.8%)	
Given	166 (79.8%)	712 (77.2%)	0.419
Adjuvant and/or neoadjuvant anti-HER2 therapy			
Not given	194 (93.3%)	810 (87.9%)	
Given	14 (6.7%)	112 (12.1%)	0.025

Abbreviations: ALND, axillary lymph node dissection; SNB, sentinel lymph node biopsy
